# A Path Less Travelled: A Case Report of an Unusual Trip of a Gall Stone

**DOI:** 10.7759/cureus.21928

**Published:** 2022-02-05

**Authors:** Surya Elangovan, Manu Vats, Sushanto Neogi, N Nasida Fathima, Vimlendra K Chaudhary

**Affiliations:** 1 Department of Surgery, Maulana Azad Medical College, New Delhi, IND

**Keywords:** cholecystectomy, enterolithotomy, cholecystogastric fistula, intestinal obstruction, gall stone ileus

## Abstract

Gall stone ileus is one of the rare complications of patients with cholelithiasis and usually affects elderly females. The usual sites for the stone to get impacted are the distal ileum and ileocaecal valve. Computed tomography (CT) remains diagnostic and surgery is the treatment of choice. A 60-year-old diabetic female, who was diagnosed with gall stone-induced pancreatitis one month ago, presented to the surgical emergency department with complaints of right upper abdominal pain with recurrent vomiting and constipation of five days duration. The patient was managed conservatively. A provisional diagnosis of subacute intestinal obstruction was kept and a barium meal follow-through (BMFT) was requested. However, BMFT was inconclusive. After two weeks, she presented again to the emergency department with clinical features of subacute intestinal obstruction. The patient was planned for exploratory laparotomy in view of recurrent episodes of obstruction and the presence of peritonism. Intraoperatively, we encountered a cholecystogastric fistula with a gall stone of size approximately 6.5x4 cm impacted at approximately 60 cm from the ileocaecal junction and dilated proximal small bowel loops. The surgical procedure comprised enterolithotomy and cholecystectomy along with repair of cholecystogastric fistula done. The patient had an uneventful postoperative course. Gall stone ileus is a rare cause of small bowel obstruction. Gall stone ileus presenting with a recent history of pancreatitis further makes the suspicion very unlikely.

## Introduction

Gall stone ileus is one of the rare complications of cholelithiasis, accounting for 0.3-0.5% of the patients with cholelithiasis [1]. The name is a misnomer since it is never an ileus but a mechanical bowel obstruction caused by the gall stone. Gall stone ileus contributes approximately 1-4% toward the causes of small bowel obstruction [2].

Gall stone ileus usually presents in elderly females (70-80 years) with a female to male ratio of 3.5 to 4.5:1 [3]. It occurs due to abnormal bilioenteric fistulous communication, allowing the gall stone to enter the gastrointestinal tract, resulting in obstruction. The abnormal communication occurs most commonly in the duodenum (65%), followed by the colon (25%), and very rarely in the stomach (15%). The usual sites for the stone to get obstructed are the distal ileum and ileocaecal valve (60-75%) [4]. When the impacted stone causes gastric outlet obstruction, it is eponymously called Bouveret syndrome, accounting for 1-4% of those with gall stone ileus [5].

A computed tomography scan remains the investigation of choice with a specificity of 100% [6]. Surgery remains the management of choice. However, controversy exists about the choice of surgery to be employed. A one-stage procedure consisting of enterolithotomy, cholecystectomy, and fistula repair is preferred in low-risk patients [7]. The mortality due to gall stone ileus is around 7-30%. The high mortality is attributed to the fact that most of the patients are elderly with multiple comorbidities [6].

## Case presentation

**Figure 1:**
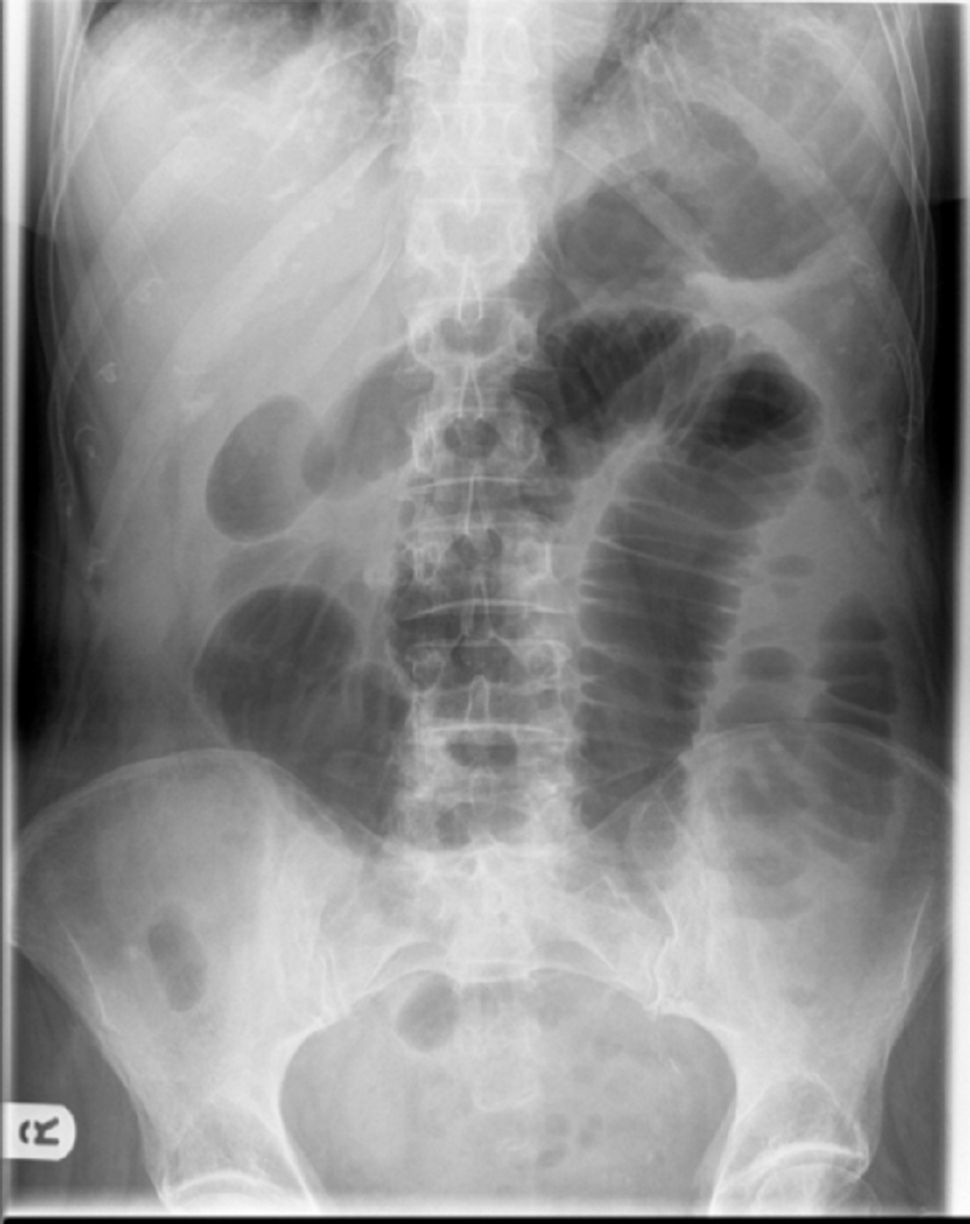
Plain abdominal radiograph showing signs of small bowel obstruction with dilated small bowel loops

**Figure 2:**
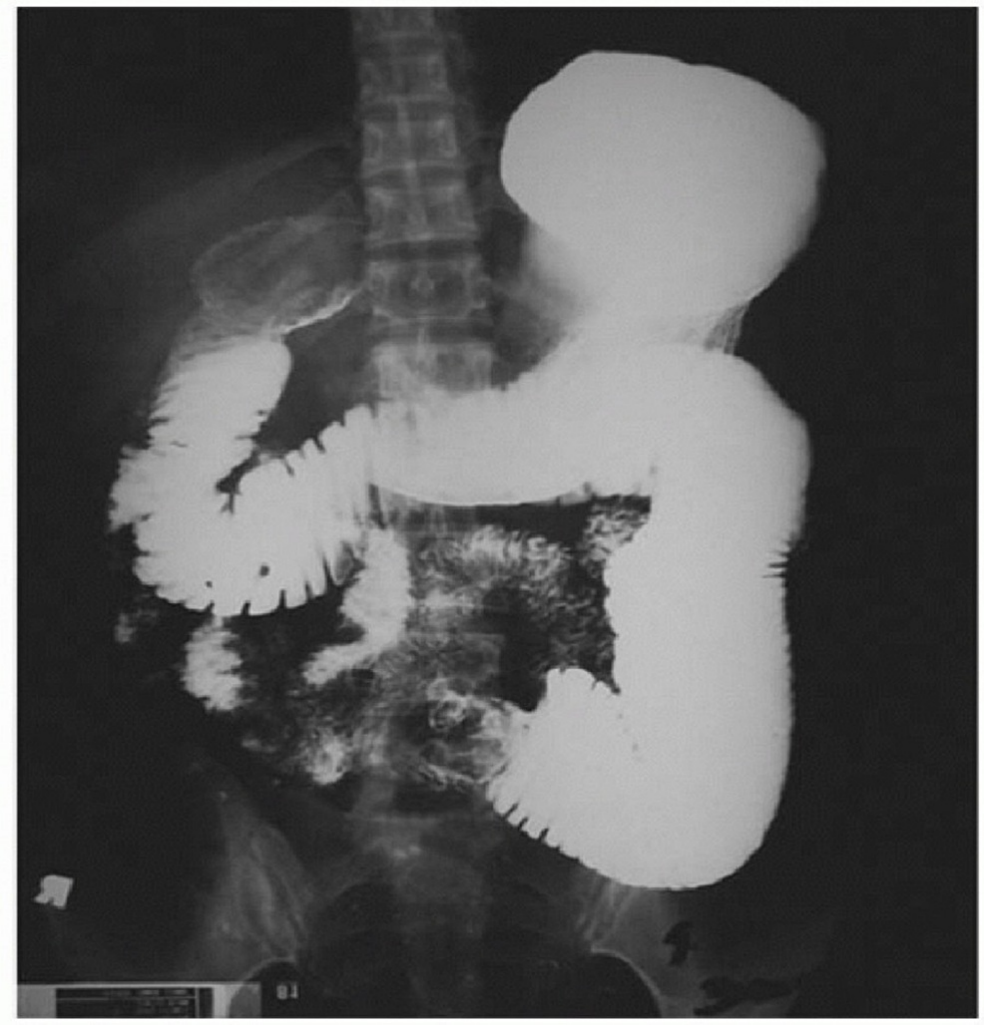
Barium meal follow-up of the patient showing luminal obstruction of the small bowel

Our patient was a 60-year-old diabetic female who presented to the surgical emergency with complaints of right upper abdominal pain with recurrent vomiting and constipation for the past five days. She was diagnosed and treated for gall stone-induced pancreatitis one month ago in a different institution. She was worked up for the same with an ultrasound abdomen and contrast-enhanced computed tomography (CECT). On review of her previous investigation records, the ultrasound of the abdomen revealed a single large calculus in the gall bladder with normal common bile duct anatomy. The CECT that was done during the episode of pancreatitis revealed acute pancreatitis and did not reveal any gall stone. The patient gave a history of being treated successfully for pulmonary tuberculosis 15 years ago. On examination, she was mildly dehydrated with a pulse rate of 105/min and normal blood pressure. The abdomen was soft, mildly distended, non-tender, with exaggerated bowel sounds. Her hematological and biochemical investigations, including amylase and lipase, were within normal limits. The abdominal radiograph revealed dilated bowel loops with few air-fluid levels (Figure 1). The patient was managed conservatively with nasogastric decompression for 24 hrs, following which she got relieved of her symptoms. A provisional diagnosis of subacute intestinal obstruction secondary to abdominal tuberculosis was made and the patient was planned for barium meal follow through to rule out tubercular stricture. Barium meal follow-through revealed dilatation of the proximal jejunal loops with delayed small bowel transit time and mucosal thickening along the mesenteric border of the dilated jejunal loops (Figure 2).

Two weeks after undergoing barium meal follow-through, the patient developed recurrent episodes of vomiting and non-passage of flatus and feces with abdominal distention. On examination, the patient was tachycardic and the abdomen showed signs of peritonism. After adequate resuscitation, erect and supine X-rays of the abdomen were done. These revealed multiple air-fluid levels with dilated small bowel loops and no pneumoperitoneum. In view of recurrent episodes of obstruction and the presence of peritonism, the patient was taken up for exploratory laparotomy.

**Figure 3:**
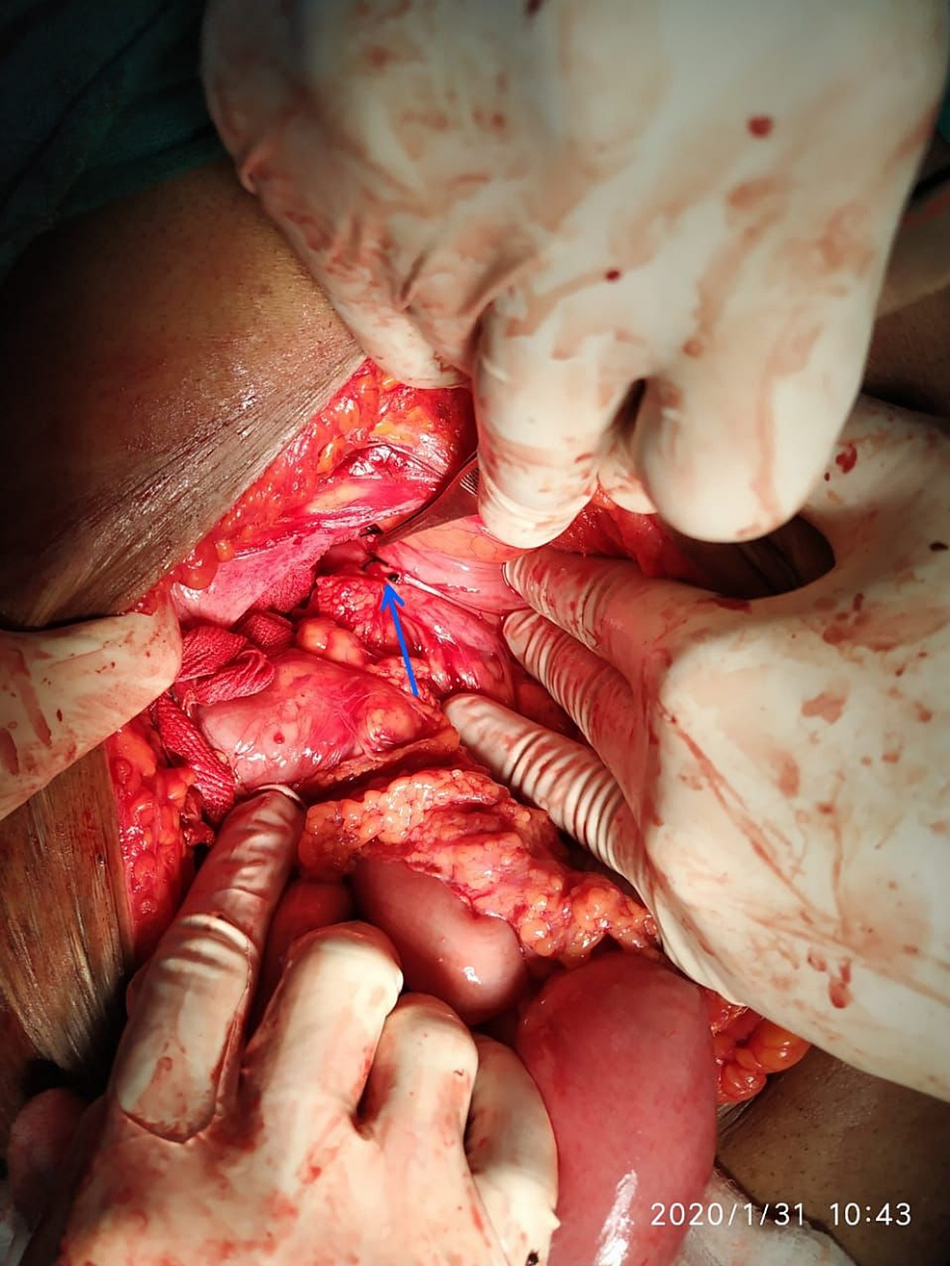
Intraoperative view of the cholecystogastric fistula shown by a blue arrow

**Figure 4:**
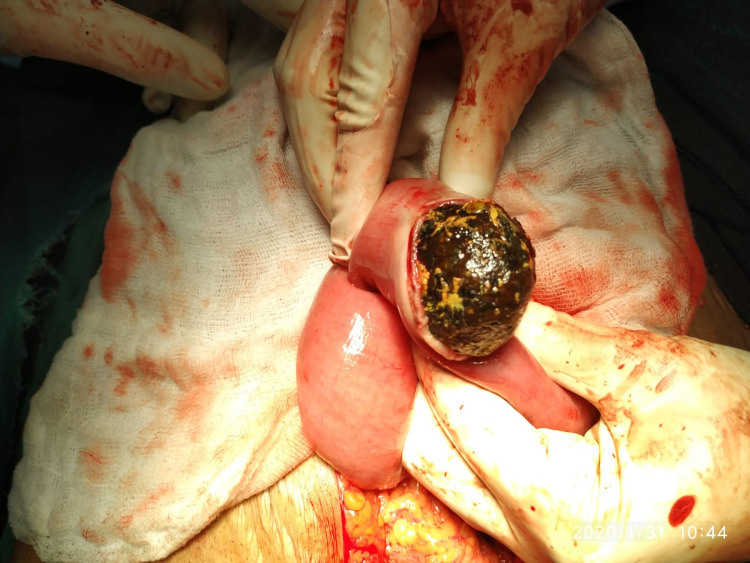
Gall stone retrieved through enterolithotomy

Intraoperatively, we encountered a cholecystogastric fistula (approximately 1x1.5 cm) (Figure 3) with a gall stone of size approximately 6.5x4 cm impacted at approximately 60 cm from the ileocaecal junction (Figure 4) and dilated proximal small bowel loops. An enterotomy was done to remove the impacted gall stone and it was closed using interrupted sutures. The cholecystogastric fistula was dismantled and the defect was repaired with interrupted sutures. This was followed by cholecystectomy. The postoperative course of the patient was uneventful.

## Discussion

**Figure 5:**
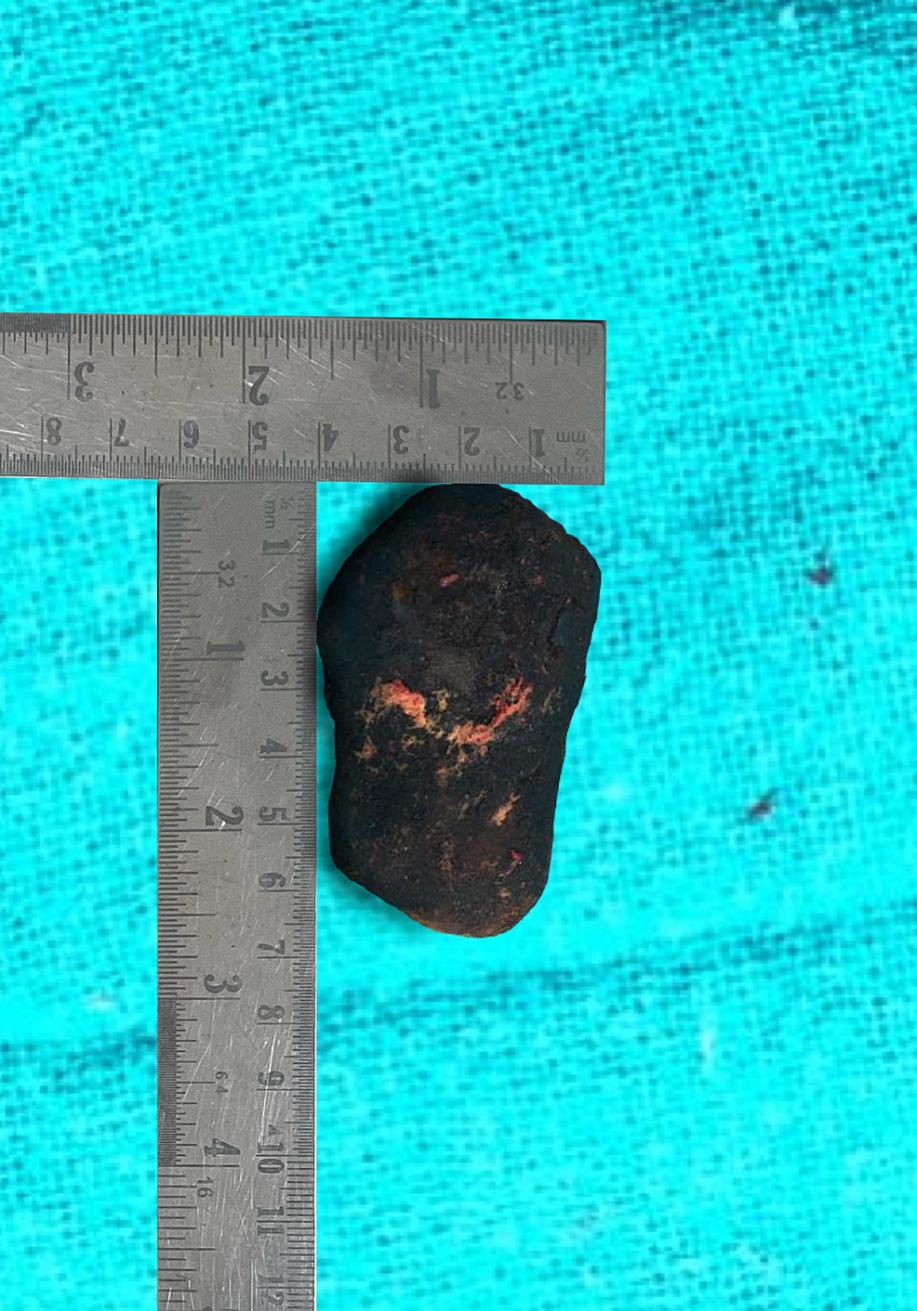
Gall stone retrieved from the distal ileum

Gall stone ileus usually presents in elderly females (70-80 years). The oldest patient of gall stone ileus who successfully underwent surgery was 91 years old and the case was reported from Japan [8]. The youngest patient with gall stone ileus was 13 years [9]. Gall stones tend to obstruct the intestinal lumen because of the presence of one or more gall stones having an average size of 2.5 cm or more. The largest reported size of stone ranged from 7 cm to 17 [10], and in our study, the size of the stone removed was 6.5 cm (Figure 5).

Patients with gall stone ileus very rarely present with gall stone-induced pancreatitis; only three cases have been published so far. However, our patient with gall stone ileus presented with a prior history of gall stone-induced pancreatitis. According to the available literature, a plausible reason for this is that the stone was causing external compression at the ampulla, as seen in the case of Bouveret syndrome, and this could be the reason for the initial presentation of the patient [11].

The most common pathophysiology of gall stone ileus is the migration of a stone through a bilioenteric fistula. Bilioenteric fistulas account for only 2% of all biliary fistulas, and they accompany gall stone disease in 90% of the cases [12]. There may be anomalous peritoneal folds between the gall bladder to the duodenum, the colon, or the stomach in that order of frequency. These anomalous folds may be associated with the gall stone ulcerating through them to cause bilioenteric fistulas [13]. The stone entering the intestine produces symptoms of incomplete obstruction, the so-called ball valve effect [14]. As in our case, the patient had an initial event of a subacute obstruction, which was relieved spontaneously followed by a second attack of acute intestinal obstruction, which could be due to the ball valve effect. The usual site for the stone to obstruct is the distal ileum and ileocaecal valve (60-75%) [4]. A more proximal obstruction is often less reported and the sites are jejunum (20-40%), stomach (15%), and duodenum (1-4%) [5].

A plain abdominal radiograph can be helpful for the following findings of the Rigler triad: 1) pneumobilia, 2) small bowel obstruction, and 3) ectopic radio-opaque gall stone. Two out of these three findings suggest gall stone ileus but the literature reports that it is seen in only 9-87% of cases [15].

The sensitivity of ultrasound, when combined with a plain radiograph, is around 74% [10]. CT is the investigation of choice in the case of gall stone ileus, as it locates the ectopic gall stone and the site of obstruction. The sensitivity is around 93% and specificity is 100% [6] but in our case, the CT scan that was done during the period of pancreatitis did not reveal any gall stone. Endoscopy can detect the obstructed stones in the stomach, duodenum, and colon. The diagnosis is often made at laparotomy in 50% of the cases, as was made in our case [15].

Surgery remains the mainstay of treatment but the choice of the best surgery is still controversial. Enterolithotomy alone is recommended for patients with sick patients and significant comorbidities (American Society of Anesthesiology (ASA) class III & IV). In this surgery, the bowel is opened longitudinally, just proximal to the site of obstruction. The disadvantages of performing only enterolithotomy include recurrent gall stone ileus (33%) and cholangitis (60%) [16].

Enterolithtotomy with cholecystectomy with fistula repair at the same setting (one-stage procedure) is preferred in patients who are hemodynamically stable at the presentation or falling in the ASA I and II groups. Enterolithotomy with delayed cholecystectomy and fistula repair is also advocated to reduce mortality and morbidity. Clavien et al. reported that the age and comorbidity matched patients had no difference in outcome among these two procedures [1]. As in our case, the patient's general condition was amenable for us to proceed with a single-stage procedure.

Laparoscopic enterolithotomy is also an available option but the conversion rate is more than 50%, as reported in the literature [17]. Endoscopic removal of stones is reserved for recurrent Bouveret syndrome.

## Conclusions

Gall stone ileus, although a rare cause of small bowel obstruction, is rarely diagnosed preoperatively. Patients with gall stone ileus presenting with a recent history of pancreatitis further make the suspicion very unlikely. A high index of clinical suspicion is required to diagnose them preoperatively. Most of the time, the diagnosis is often established after a laparotomy, irrespective of an inconclusive investigation. Despite no definitive preoperative diagnosis, it is justified to proceed with the laparotomy, as the delay hampers the outcome of the surgery. The choice of surgery is based on the evaluation of the patient’s condition and the surgeon’s intraoperative judgment.
